# Identifying who adolescents prefer as source of information within their social network

**DOI:** 10.1038/s41598-023-46994-0

**Published:** 2023-11-20

**Authors:** Scarlett K. Slagter, Andrea Gradassi, Anna C.K. van Duijvenvoorde, Wouter van den Bos

**Affiliations:** 1 Department of Developmental Psychology, University of Amsterdam, Amsterdam, 1001 NK The Netherlands; 2 Amsterdam Brain and Cognition, University of Amsterdam, Amsterdam, The Netherlands; 3 Department of Developmental and Educational Psychology, Institute of Psychology, Leiden University, Leiden, The Netherlands; 4 Leiden Institute for Brain and Cognition, Leiden, The Netherlands; 5 Max Planck Institute for Human Behavior, Berlin, Germany

**Keywords:** Psychology, Human behaviour

## Abstract

Adolescents are highly influenced by their peers within their social networks. This social influence can stem from both unsolicited peer pressure and the active search for guidance. While extensive research examined the mechanisms of peer pressure, little is known about who adolescents prefer as a source of information. To address this gap, we conducted two independent studies using a novel social search paradigm that allows participants to choose which social sources they wish to observe. In both studies, adolescents demonstrated a preference for their friends over non-friends, as well as for peers who were perceived as trustworthy. Across both studies, we found mixed evidence for the role of perceived popularity as a selection criterion. Notable, study 2 revealed the significance of “cool”, “admirable” and “acting mean” as additional characteristics of preferred peers, traits that are often associated with elevated peer status. It also revealed an interest for peers perceived as being smart. These findings highlight the active role adolescents have in choosing social sources and emphasize the importance of multiple peer characteristics. Future research should investigate whether adolescents’ interest in these types of peers is contingent upon specific social contexts, age groups, and peer cultures.

## Introduction

When uncertain about what to do, people often search for information. One of the most accessible sources of information is our direct social environment. Peers have shown to influence adolescents’ behaviour in various ways (e.g. by demonstrating specific behaviours or by stating explicit opinions^1,2^, for better^3,4^ or worse^5,6^. In adolescence, social influence is often negatively portrayed as undesirable and unsolicited, where adolescents are represented as passive receivers of peer pressure. More recently, social influence has also been viewed as functional, where peer conformity can be seen as a tool to promote affinity, group identity, and positive behavioural change^1,4,7–11^. Although this provides a more comprehensive view on social influence, it still does not account for the fact that adolescents are not only passive receivers of peer influence. Adolescents also actively seek out opportunities to observe others’ behaviour or ask for advice, to guide their decisions^12–14^, and thus play an important role in shaping what information they receive. This is a crucial aspect in the social influence process, because whether observing the choices of others (referred to as “social information”) is beneficial depends on its source^15^. For example, consulting daredevil peers may stimulate risk-taking behaviour, whereas risk-averse peers may reduce such behaviour^16^. Recent studies showed that, in adolescence, social information search and its use is particularly prevalent in uncertain situations when facing incomplete information^12,17,18^. In peer groups, uncertainty about how to behave might frequently occur as they face novel situations for which group norms are not directly observable, and thus adolescents seek out (social) cues for how to behave. However, very little is known about who adolescents seek to observe.

Multiple lines of work highlight that friends are a key source of social influence. Numerous empirical studies showed a considerable influence of friends on adolescent’s behaviour in a wide range of behaviours such as substance-use, antisocial but also prosocial behaviour (for example^19–26^). According to social learning theory, individuals prefer to model friends (copy-friends strategy^15^) since social learning is more effective when the demonstrator and observer experience the same environment and seek the same behavioural goals or preferences^27^, which is the case with friends^28^. As they share the same environment and goals, friends are more likely to provide reliable information^29^. In line with this theory, adolescents reported to feel more similar to friends, and this similarity has shown to moderate the extent of peer influence^30^. Another motive to model friends could be adolescents’ desire for a sense of belonging within their in-group; resembling the behaviour of their friends will increase the affiliation with these companions^9,28,31^. All these findings suggests that adolescents would seek guidance from friends and similar individuals, especially when facing uncertainty in their own preferences^32^.

Adolescents might also be motivated to observe the preferences of popular peers. Previous studies have shown that popular peers are influential in the domains of antisocial and risky behaviours^33,34^, as well as prosocial behaviour^1,3^. In these studies, early and mid-adolescents conformed more to decisions made by high-status peers (i.e., high in likability and/or perceived popularity) than those made by low-status peers^3,33–35^. Heightened sensitivity to the behaviour of popular peers has been explained by adolescents’ need to increase and maintain their own social status and their need to belong to this group^36–39^. Popular peers might be seen as socially skilled individuals who signal which norms and behaviours are socially desirable and successful, helping the adolescent to prevent social rejection and learn about how to obtain status. It is therefore likely that adolescents are focused on observing popular peers as well, especially for status-related behaviours (such as risk-taking) and preference-based judgments.

The importance of friends and popular peers as source of information might change with age. In early adolescence, the need to seek out popular peers might peak as in this stage adolescents need to find their social position due to their school transition (and thus social group). When transitioning to a new social group, uncertainty arises about prevailing norms and one’s own sense of identity. Over time, as adolescents’ quality and depth of friendship develops^40,41^ , they become more similar to their friends and might grow more secure in their identity^9,39,42^. So far, only few relevant developmental studies exist, making it difficult to generate strong hypotheses on how the role of friends and popular peers as source of information might change across adolescence. One previous study reported that being part of a popular group was more important for early and mid-adolescents (aged 12–16 y) than for late-adolescents (aged 16–18 y)^43^. Another study suggests that the priority given to popularity in contrast to the priority given to friendship, starts to decline in emerging adulthood (> 18 years)^44^. Based on the described changes in social context and individual development, together with these two studies, we hypothesized that, at least in early adolescence popular peers would be consulted more often than non-popular peers and that, if anything, this effect would decrease toward late adolescence. In addition, we hypothesized that adolescents’ focus on friends compared to non-friends would increase toward late-adolescence.

Although passive peer influence in adolescence has been extensively studied, we have little knowledge about whom adolescents *choose* to observe in their classroom, when having access to the behaviour of numerous sources. In previous experimental studies, adolescents saw the choices of specific peers pre-selected by the experimenter. While this setup allows us to accurately measure the impact of specific peers, it may over- or underestimate the importance that those sources have in everyday decisions because it bypasses the question whether those peers are consulted in the first place, and whether they can compete with other available sources. For instance: high status peers may induce higher conformity rates than low status peers (e.g.^33,34^), but how often do adolescents consult high status peers, relative to their friends, in real life? Investigating who adolescents prefer as social source will provide a better understanding of which peers are important for guiding adolescents’ behaviour. Furthermore, it may reveal the importance of other characteristics than popularity or friendship that received little attention so far^39^.

**Figure 1:**
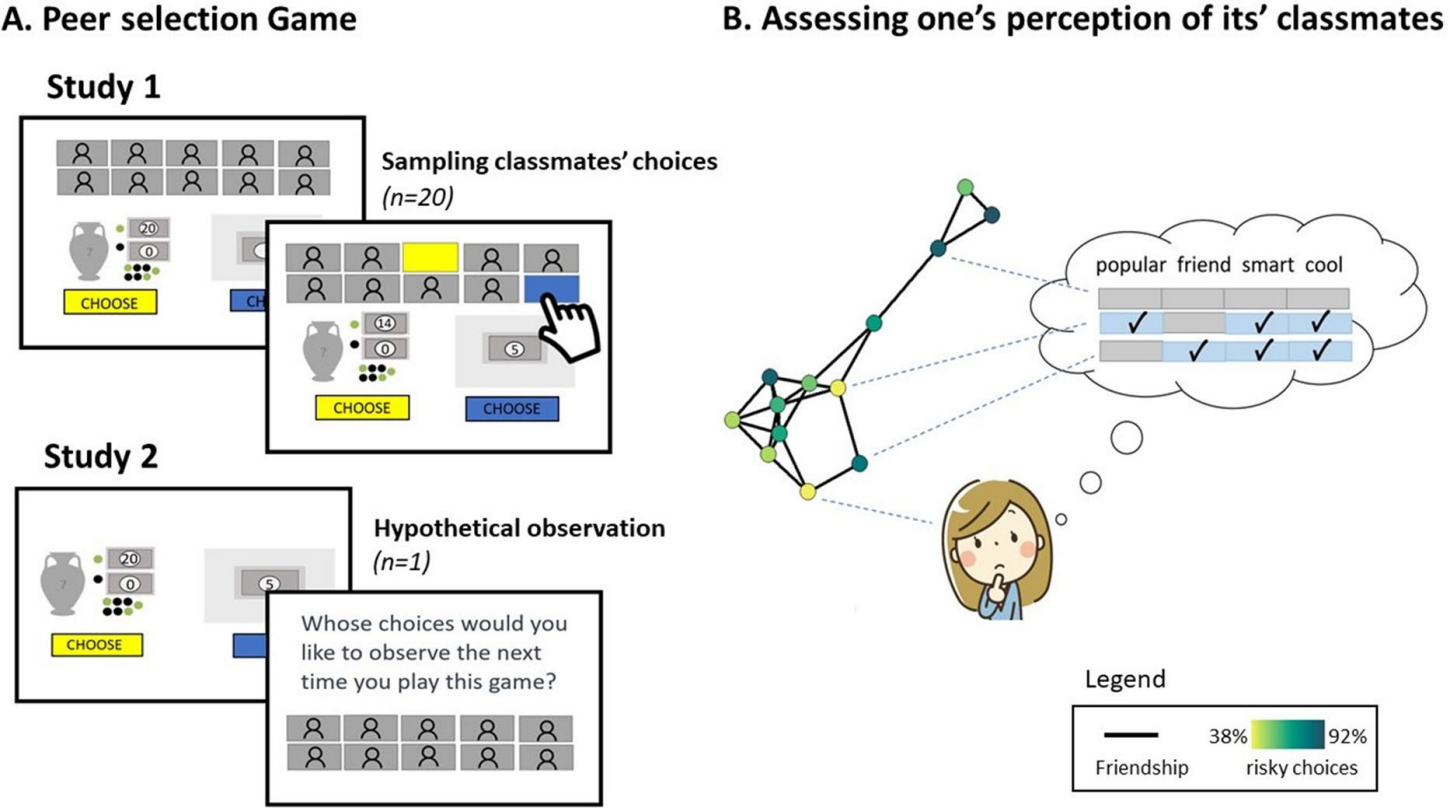
General set-up of study 1 and 2, to examine the peer characteristics of selected peers for choices made under uncertainty. (**A**) Study 1 consisted of two sessions. During the first session, participants played a decision-making game alone over 20 trials. In all trials participants had to choose between a sure option (blue; worth 5 points) or a risky option (yellow; a random draw from a gambling vase). The risky option resulted in 0 points, if a black ball was drawn, or an amount of either 8, 14, 20, 32 or 50 points (depending on the trial), if a green ball was drawn. In the follow-up session, participants selected the classmates of whom they wanted to see the choices, prior to making their own decision. Classmates’ real-made choices, obtained from the first session, were presented as covered boxes in a matrix (social board). The participant could reveal these choices by clicking on the grey boxes, which were labelled with the classmates’ names to indicate the origin of the choice. In study 2, participants played the same gambling game, but were asked at the end of the game hypothetically which classmates they wanted to observe when they would play the gambling game again. (**B**) To identify what type of peers participants did (not) select in the game, we used a peer nomination questionnaire. Participants nominated their classmates on various peer characteristics (e.g. smartness, popularity, trustworthiness) in the first session. Friendship nominations were also used to construct the classroom social network. Here, an example social network from one of the participating classrooms shows how participants (i.e. nodes) are connected (i.e. lines) within their classroom based on a mutual nomination of friendship by both parties. Node colour reflects the percentage of risky choices each classmate made in the game.

In this paper, we present two independent studies on how adolescents’ search for social information in their school social network. In the first pre-registered study (https://osf.io/bz2en; *N* = 173, ages 11–18 y), we combined peer nominations with a novel social search paradigm, to address which type of peers adolescents consult before making decisions under uncertainty in an incentivized gambling game (see Fig. 1A,B). In the game, participants could reveal the previous choices (i.e. risk preference) of their classmates, by selecting their names on a board, prior to choosing between a fixed payoff or a gamble. The peer nomination questionnaire enabled us to identify how the selected classmates were perceived by the participant (e.g., trustworthy). Our design allowed us to test the hypotheses that friendship and popularity are both important selection criteria. Moreover, with a variable selection procedure, we could explore the importance of other peer characteristics in predicting peer selection. The second study (N = 278, ages 12–17 y) was a conceptual replication of study 1, and explored a wider range of peer characteristics linked to the broad concept of status and popularity (see Fig. 1B). In contrast to study 1, participants did not reveal choices of their peers, but only reported which classmates they wanted to consult when they would play the gambling task again in an upcoming session (Fig. 1A).

## Study 1: Monitoring real-time peer selection for choices under uncertainty

### Methods

#### Participants

Ten classes from two Dutch secondary schools took part in our study, resulting in a total sample of 173 participants (56% female, M age (SD) = 15.1 (1.62). Within the Dutch school-system, adolescents follow secondary education in groups of approximately 20–30 students, and these student cohorts remains nearly the same throughout the duration of their secondary education (depending on the education level, this varies between 4 and 6 years). Our class-based setup allowed us to target adolescents and their classmates at school. Here, classmates engaged as participants but also functioned as social sources for other participants within our experimental task. Participants age ranged from 11 to 18 years and represented three different grade-levels (1st, 3rd and 5th grade), with 91% following pre-university secondary education and 9% preparatory vocational secondary education. Participants who were not able to select at least one friend and one popular peer on the social consulting board of the gambling game were excluded from the main model analysis (see Table S1 in Supplemental Materials), resulting in a final sample of 140 participants (57% female, M age (SD) = 15.05 (1.59); see Supplemental Material Fig. S1 for the age distribution).

#### Procedure

Data was collected in two sessions which had a similar set-up. Both sessions took place at Dutch high schools in the regular classroom setting. Tables were set apart and screen dividers were provided on each table, to increase one’s privacy and comfort in performing the experiment alone. Participants performed a battery of questionnaires and games on a tablet. Each session lasted approximately 45 min, with a 2 to 3-week interval between sessions. Session 1 aimed to collect the solo choices of each participant and its classmates, in a decision-making game under uncertainty. In session 2, these classmates' choices were used to develop a social search paradigm tailored to each participant. Session 2 aimed to record which peers were selected for information during the game. Classes received a monetary incentive for each session (5 euro per participant). In addition, participants were instructed that they could gain lottery tickets by playing three behavioural games (part of a larger study), to incentivize performance. Collected lottery tickets were used to select one participant per class for a €40 online shop voucher. All participants gave informed consent prior to data-collection. For minors, we additionally received informed consent from their parents or legal guardians. All procedures were approved by the Ethics Review Board Faculty of Social and Behavioural Sciences of the University of Amsterdam (case number 2019-DP-10269) and performed in accordance with relevant guidelines and regulations. We made sure that all participants were aware of the possibility that their choices, made in session 1, would be viewed by others.

#### Assessing the characteristics of peers

At the beginning of the study, participants completed a peer nomination questionnaire designed to map different characteristics of each classmate, based on the participants’ own rating^45^. Participants could choose to nominate their classmates for different type of characteristics (see Table 1 for the list of items). Participants could select as many names as they wanted, or the option ‘nobody’ for each item. This resulted in a binary coding (1 = yes, 0 = no) for each classmate dyad, indicating whether the classmate was nominated for a certain characteristic by the focal participant. Thus, only nominations from the focal participant were used to determine the perceived characteristics of each classmate.

**Table 1:** Inclusion of peer nomination items for study 1 and 2.

Peer characteristic (question-item)	Study 1	Study 2
Friend (*Who are your friends?*)	x	x
Most popular *(Which classmates are most popular in your class?)*	x	x
Kind *(Which classmates do you like?)*	x	x
Not kind *(Which classmates do you not like?)*	x	x
Trustworthy *(With whom would you share a secret or your feelings (e.g. that you are in love))*	x	x
Influential *(Which classmates influence others to do what they want?)*	x	x
Leader *(Which classmates often take the lead in your group/class?)*	x	x
Smart *(which classmates are smart?)*	x	x
Prosocial *(Which classmates help you with problems (e.g. homework, fixing your bike, cheer you up))?*	x^1^	
Advisor *(which classmates can give good advice to others?)*	x^1^	
Bully *(which classmate bullies others?)*	x^1^	
Best friend (Which classmate(s) is/are your best friends?)		x
Admirable *(Which classmate do you look up to/ do you admire?)*		x
Cool *(Which classmates are cool?)*		x
Mean *(Which classmates are mean? (e.g. a classmate who bullies, excludes or humiliates someone)*		x

All characteristic-items were answered with unlimited nominations from a checklist with all class members. ^1^Items that were of interest for the overarching study but not for this specific study were replaced in study 2 for characteristics, that have been associated with popularity.

#### Assessing the network distance between peers

Reciprocal friendship nominations (obtained from the peer nomination questionnaire) were used to build a representation of the social network for each classroom. In those network representations, nodes are peers within a classroom, and a tie is formed when both peers nominated each other as friend (undirected friendship network^30^). In this study we were interested in the network distance between peers, as an index measure of closeness. The network distance is a measure of how many ties separate two peers within a social network^46–48^. Therefore, if two peers have a distance of 1, it means they are friends; when network distance = 2, the peers are friends of friends (and thus connected through a mutual friend), and so on (see Fig. 3A). Network distance was treated as a categorical variable, as we assumed that the degree of change between each successive pair of distance is not identical. Representations of the friendship networks were created with the R package *igraph*^49^.

#### Social search paradigm for choices under uncertainty: monitoring real-time peer selection

We developed a novel social search paradigm to investigate who adolescents select for social information prior to decision-making under uncertainty. In this paradigm, participants had the option to gather information about the choices of their classmates before they chose between an option with a sure (i.e. safe) or an uncertain (variable, thus, more risky) outcome (see Fig. 1A and Supplemental Material Fig. S2B). In all of the 20 trials, classmates’ choices (collected in session 1) were hidden under covered boxes (i.e. ‘the social board’), which could be revealed by clicking on the boxes. The search was voluntary, and each box was marked with the classmate’s name that allowed us to explore which type of peers were selected for information. Once a box was clicked, it would reveal which choice this peer made; yellow indicated the uncertain, more risky option, and blue indicated the sure option (see Fig. S2B in Supplemental Materials). With this paradigm, we mimicked the way in which adolescents can freely observe the behaviour of all their classmates in the classroom setting.

Our social search paradigm included the gambling game, originally designed to measure one’s preference under risk and uncertainty^12,50,51^. In this game, the sure option had a fixed payoff of 5 points. The gambling option entailed drawing a ball from a gamble vase containing green and black balls. Drawing a ball from the urn could result in a higher gain amount (green ball) or in winning nothing (black ball). Instead of showing the full distribution of green and black balls, participants were only shown a sample of 7 (out of 100) balls. The goal of showing an ambiguous sample was to induce more uncertainty about the outcome and therefore making social information probably more appealing for participants^2,18^. Whether it is worth gambling depended on one’s individual preference for risk, and thus participants were instructed that there were no right or wrong answers. Participants were instructed that three trials would be randomly selected by the computer and played out to determine the participant pay-off. Collected points were converted into lottery tickets for a raffle price of €40. The game was developed with the software NEUROTASK (https://scripting.neurotask.com).

The experimental nature of this game came with various advantages. First, the game involved choice dilemmas in which participants and their classmates, who served as possible sources, had no prior knowledge or experience. Therefore, there was no need to control for the participants’ prior knowledge or expertise, that otherwise could have contaminated peer selection. Most importantly, we wanted to share real choices of classmates without potentially harming the social relationship between classmates, which could happen when sharing personal statements about certain behavioural actions (i.e. personal injunctive norms about alcohol or drug use). Additionally, no feedback was given about the outcome of each decision, so participants could not track the outcome of their own and classmates’ choices (thus, in hindsight, they would never know whether it was a good idea to take a risk or not). This was to limit 1) ethical concerns on sharing classmates’ choices, and 2) spillover effects on sampling for subsequent trials. Finally, the decisions made in this game resulted in real payoffs, thus obtaining and following the choices of others had the potential to benefit one’s payoff (no cheap talk).

### Statistical analyses

For each participant, we calculated how often each classmate was selected for social information within the searched trials (i.e. selection rate). The frequency of selection was divided by the total number of searched trials. This resulted in a selection rate between 0 and 1 for each classmate, that was presented on the social consulting board. Note that classmates who refrained from participating were not presented on the social board, and were thus opt out as a selection option. All statistical analyses to predict the peer selection were conducted in R statistical software, Rstudio v. 1.3.1093. Unless stated otherwise, analyses were performed with the *lmer* function of the *lme4* package^52^. All models included a random intercept per participant to account for individual differences in peer selection.

#### Peer selection—main models

A logistic mixed-effects model was used to predict the selection rate of each peer. The binary variables ‘perceived as friend’ and ‘perceived as most popular’ were used as predictors. These predictors were added as an interaction term with age, to test for the age-dependency of these peer characteristics (see Supplemental Material Table S1, Model Study 1a). In a subsequent analysis, this main model was updated by exchanging perceived as friend with the estimated network distance as predictor (see Table S1, Model Study 1b in Supplemental Materials), to investigate whether friends of friends are also more likely to be chosen for social information, next to friends only. The fit of both models was evaluated with the Akaike Information Criterion (AIC) and Bayesian Information Criterion (BIC) to assess whether model fit improved (indicated by lower values for AIC and BIC). We excluded participants who were not able to select a friend or someone who they perceived as popular on the social board of the game, due to non-participation or absence of these classmates. This resulted in a subsample of 140 participants for our main model of study 1 (M age (SD) = 15.05 (1.59), 57% Female).

#### Best predictors for peer selection–exploratory model

Exploratively, we were interested in finding the best set of predictors to describe selected peers. This was done by applying a Lasso-type approach for variable selection (L1-penalized algorithm) on a logistic mixed model with all measured peer characteristics (see Table 1). In contrast to step-wise variable selection, this automatic approach starts with the most complex model and actively eliminates predictor variables by setting coefficients of less contributing predictors to zero. While both approaches aim to find the best model, Lasso forces reduction of variables to reduce overfitting and minimize predictor error. This also constrains the model’s complexity for interpretability^53,54^. First, the most optimal shrinkage parameter for the Lasso model was established by running the model for 100 iterations with different values for the shrinkage parameter (ranging between 0 and 500). Finally, the shrinkage parameter resulting in the model with the lowest AIC-value was used to shrink the number of predictors of the full model. This statistical analysis was performed on the full dataset (N = 173) using the *glmmLasso* package^55^.

### Results

#### Adolescents search for social information

From the 173 participants, 95% of the participants (N = 164) searched within the gambling game by revealing a choice of at least one classmate. These participants searched on average in 54% of the trials (*M* = 10.76 trials, *SD* = 5.80), and the mean number of peers consulted on search trials was 12.34 (*SD* = 8.97; see Supplemental Material Fig. S3 for a distribution of the search behaviour). This shows that adolescents were often interested in seeing what their peers did. Further exploratory analysis showed that participants were influenced by the sampled information from their classmates; higher proportions of sampled choices favoring risk increased the likelihood of choosing risk by the participant as final choice (see Supplementary materials Sect. 1, for more details).

#### Friends, but not popular peers, are consulted more often for information

**Figure 2:**
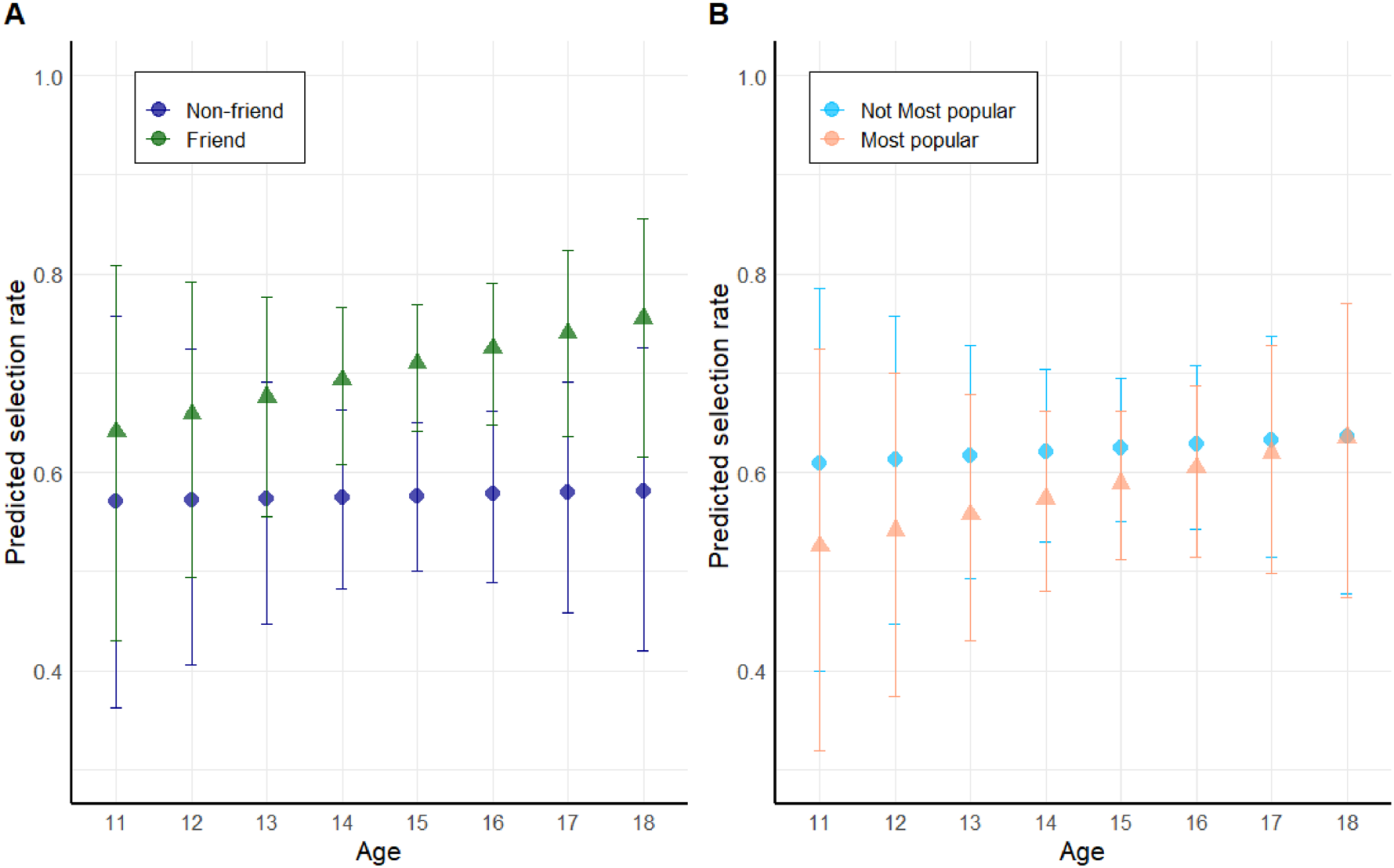
Predicted selection rate based on the model fit estimates across age for friends and most popular peers. (**A**) Predicted effect of friend (compared to non-friend). (**B**) Predicted selection rate for peers nominated as most popular contrasted against peers not attributed with that characteristic. Dots represent the estimated selection rate with 95% confidence intervals (CI) per age category.

First, we examined whether friends and popular classmates were more often selected as social source compared to non-friends and non-popular peers. We applied a mixed effects logistic model with friend (0 = no, 1 = yes) and most popular (0 = no, 1 = yes) to predict the observed selection rate of each classmate (ranging between 0 and 1) within the trials that the participants searched for social information (see Methods; statistical analyses for more details). As expected, friendship was a significant predictor for peer selection. Friends were 1.80 times more likely to be chosen as social source than non-friends (logistic mixed model, main-effect of friend, Odds Ratio (OR) = 1.80, 95% CI = (1.68, 1.93), see Table 2). Selected peers turned out to be friends in 70% of the cases (see Supplemental Material Fig. S4). In addition, friends are even more likely to be selected compared to non-friends with increasing age (OR = 1.12, 95% CI = (1.05–1.20), see Table 1 and Fig. 2a). However, in contrast with our expectations, popular classmates were significantly less likely to be selected than non-popular classmates (OR = 0.85, 95% CI = (0.79, 0.91)), an effect that diminished with age (Z = 2.15,* p* = 0.03; see Table 1 and Fig. 2b). Follow-up inspection showed that the selection rate for non-popular and popular peers became more similar with age, showing hardly any difference between the selection rates for popular and non-popular peers at older ages (see Fig. 2B).

**Table 2:** Outcome of the main model of study 1.

	Dependent variable: selected peer (proportion of times selected in searched trials)
(intercept)	1.41 (1.03–1.92) Z = 2.14,* p* = 0.033
Friend [= yes]	1.83 (1.71–1.96), Z = 17.19,* p* < 0.001
Most popular [= yes]	0.87 (0.81–0.94), Z = -3.58,* p* < 0.001
Age (continuous)	0.99 (0.73–1.35), Z = − 0.05,* p* = 0.961
Friend × Age	1.12 (1.05–1.20), Z = 3.48,* p* < 0.001
Most popular × Age	1.08 (1.01–1.16) Z = 2.15,* p* = 0.032
Number of participants (N classes)	140 (10)
Number of observations	2725

A linear mixed-effects model was fitted to the observed proportion of times that a peer was selected in the game, using ‘participant’, nested in ‘classes’ as random intercept. The number of searched trials per participants were entered as weight in the model, giving more weight to observed selection rates of participants that searched more. Model 1 tested whether the selected peers were friends or popular peers, and whether the selection of friends and popular peers depended on age. Model 2 exchanged perceived friendship for network distance. Odds ratios are given for the specific characteristics. The 95% confidence intervals (CI) are in parentheses, followed by the Z-statistics and* P* values.

#### More socially distant peers are less often observed for information

**Figure 3:**
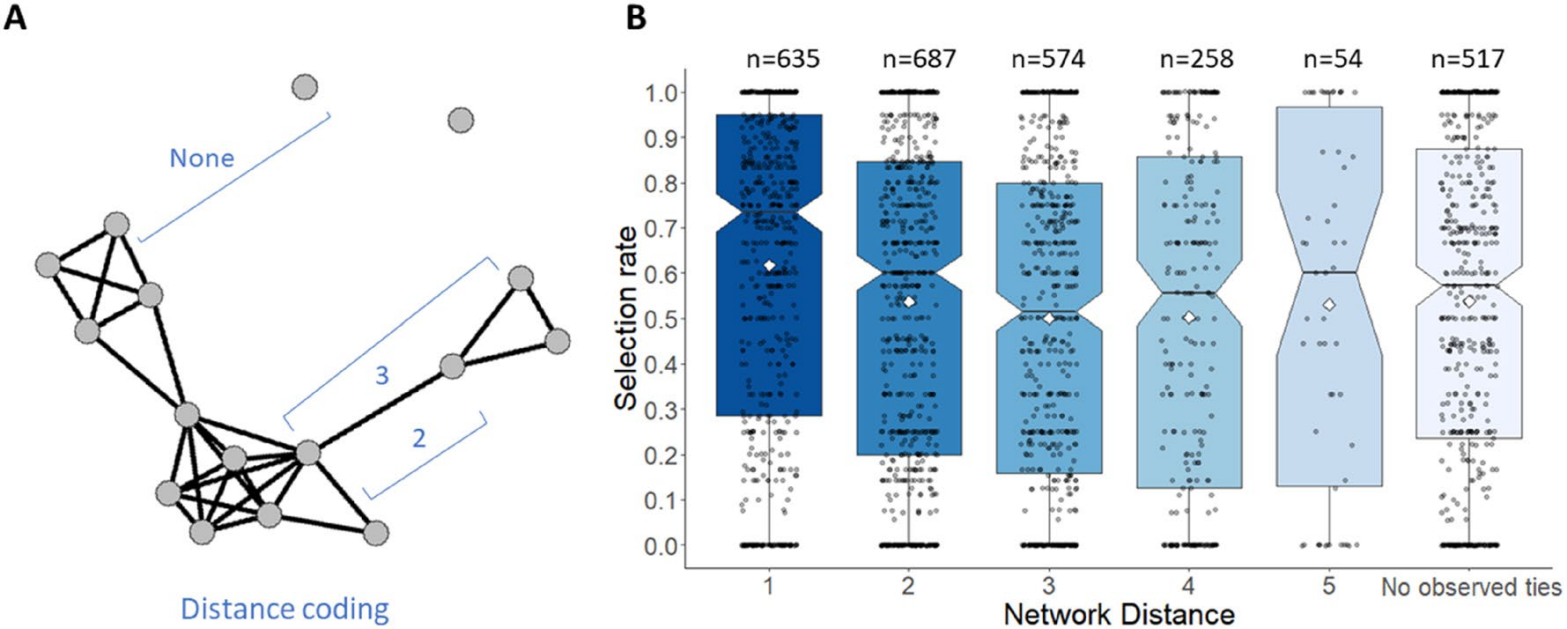
Selection rate for peers grouped on network distance. (**A**) Example of a sampled classroom network based on friendship nominations, visualizing different network distances between nodes (i.e. classmates) based on the number of ties that separate these nodes. (**B**) Selection rate is highest for friends (network distance = 1), and decreases for peers further away in the classroom network. Black bars and notches indicate the median, the white diamonds the group mean selection rate and the boxes the interquartile range. Grey dots indicate the mean selection rate for an individual classmate for each participant. The number of observations for each network distance is displayed above the box plots.

To further investigate the role of affiliation in social search, we further examined the role of network distance, by using the path length between dyads of peers within the friendship network (see Methods for variable explanation). For this analysis we substituted perceived friend (friend vs. non-friend) for the network distance measure, for which we observed the levels 1, 2, 3, 4, 5 and no tie. Here, each level refers to the number of ties that separate two peers and ‘no tie’ reflects no observed connection between two peers (see Fig. 3a). Model comparison showed that network distance improved the model fit, compared to the model with friendship as binary predictor (∆ AIC = 102, ∆ BIC = 55 in favour of model 2). In line with our expectations, the selection rate for friends of friends was higher than for those more distal in the network (see Supplemental Material Table S2), with the lowest likelihood of selecting a peer when the peer network distance = 3 (see Fig. 3B). On average, participants consulted friends in 62% of their sampling time (distance = 1), friends of friends (distance = 2) in 54% of the time, and peers related to the friends of their friends (distance = 3) in 50% of the cases. For an elaboration on the quadratic pattern of the network distance effect as seen in Fig. 3, see the Supplementary Results (Sect. 2). This likely reflects error estimates in distance 4 and above due to missing data (see Supplemental Results: Sect. 2 in Supplemental Materials). Robustness check indicated that removal of three classes with a participant rate below the recommended 70% to construct friendship networks (see Supplemental Material Table S3 for the participant rates per class^56^) did not change parameter estimates or model comparison results meaningfully (see Table S2 in Supplemental Materials).

#### Adolescents also select trustworthy peers

Exploratorily, we ran a linear mixed model with all the possible peer characteristics (see Table 1 for a full list) to explore additional peer characteristics that contribute to the selection of a source. Variable selection was determined by a L1-penalized Lasso algorithm (*glmmLasso* package^55^), developed for complex mixed models. The best fitting model based on AIC indicated the following peer characteristics as most important predictors for being selected as source: trustworthy for sharing feelings and secrets (b = 0.50, SE = 0.16, 95% CI (0.19, 0.81), Z = 3.10,* p* = 0.002) and friendship (b = 0.40, SE = 0.12, 95% CI (0.16, 0.64), Z = 3.43,* p* < 0.002). Thus, peers that adolescents select most as social source were peers perceived as trustworthy and/or friends (see Supplemental Material Fig. S5 for the correlation strength between these characteristics; *r*(3378) = 0.08–0.53). For additional details regarding the estimated coefficients of all peer characteristics, and the fit of all models across the applied shrinkage range (lambda[0–500]), see Supplemental Material Fig. S6.

## Study 2: Observation Question for choices under uncertainty

In study 2, we aimed to conceptually replicate our findings from study 1, and to test a wider range of peer characteristics, that have also been linked to the broader concept of popularity. In this second study we also aimed to confirm the effect of trustworthiness on the selection of peers, found in study 1. Study 2 followed the same at-school procedure as described above. More details on the updated design and statistical analyses, based on study 1, can be found below.

### Method

#### Participants

Twenty-two classes from two Dutch high schools (including pre-university until preparatory vocational education) were recruited. This resulted in a sample of 278 participants (54% female, M age (SD) = 14.12 (1.17); ages 12–17 y; see Supplemental Material Fig. S7 for the age distribution) from the 1st and 3rd grade. Participants followed pre-university secondary education (8%), higher general secondary education (6%) or preparatory vocational secondary education (32%), with the majority of the participants following a mixed education program (55%) for which the education level is defined after grade 3. On average, the participation rate per classroom was 56%. However, the different set-up of study 2 enabled us to include every classmate as social source (see Method section: Observation Question for the gambling game).

#### Assessing the characteristics of peers

Popularity has shown to be a broad concept with many different facets^57^. For instance, popular peers have been associated with positive attributes such as likability, but are also linked to more negative features such as dominance^58–60^. We therefore included additional items that address elements of peer status that have shown to be related to popularity (see Table 1 for the complete list and the comparison to study 1). Identical to study 1, participant’s individual nominations were used to assess how each participant perceived their classmates.

#### Observation Question for the gambling game

Participants from this study played the solo version of the gambling game in session 1 (see Supplemental Fig. S8) and were informed that they would play this gambling game again in a consecutive session. Participants were asked at the end of their solo play of the gambling game of whom they wanted to see the choices when they would play the game again in the consecutive session (see Fig. 1B). Participants were able to select all their classmates, or select ‘nobody’. There was no limit on the number of classmates participants could select. Compared to study 1, this set-up had some advantages. As the data was collected in session 1 and no sampling of real choices was involved, participants were able to select all classmates (also the non-participating ones) as source. Moreover, this set-up eliminates the effect social information (i.e. choices of others) can have on deciding to continue searching or not^12^; The immediate reveal of the peer’s choice could possibly lead to a shorter (e.g. in case of consistent social information^12^) or longer search for peer choices (e.g. in case of inconsistent information^12,61^). Thus, the sampled information could have masked the selection of potential interesting peers in some decision trials of study 1.

#### Statistical analyses

The confirmatory logistic mixed model for predicting peer selection (see Supplemental Material Table S1, Model Study 1a) was updated by adding trustworthiness and smartness as predictors (see Supplemental Material Table S1, Model Study 2). In this model, the outcome variable was 1 (= selected) or 0 (= no selection), indicating whether a classmate was chosen for observation by the focal player. Next, we ran a Lasso-regularized model for variable selection (see Methods study 1), with all the possible peer characteristics (see Table 1).

### Results

#### Replication of the role of friendship and trustworthiness

**Figure 4:**
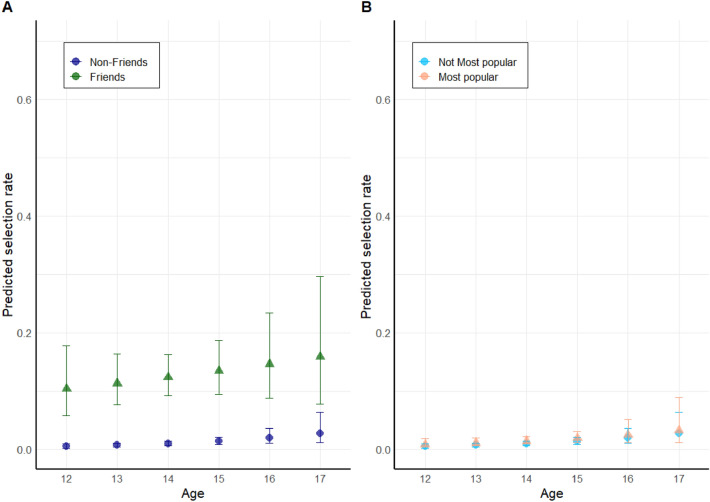
Predicted selection rate across age for friends and most popular peers for sample 2. (**A**) Predicted effect of friend (compared to non-friend). (**B**) Predicted selection rate for peers nominated as most popular contrasted against peers not attributed with that characteristic. Dots represent the estimated selection rate with 95% confidence intervals (CI) per reported age.

Of the 278 participants, 44 participants refrained from consulting a peer. The subset of 234 participants who were interested in consulting their peers selected on average 4.4 peers (*SD* = 5.6, range 1–31; see Supplemental Material Fig.S9 for a distribution of the search behaviour). Thus, most participants were interested in learning about their peers’ choices, but generally selected fewer sources compared to study 1. As expected, peers who were selected as friend or trustworthy were respectively 14.08 and 7.22 times more likely to be chosen as social source compared to those not nominated as friend or trustworthy (see Table 3 for the complete logistic mixed model results). However, in contrast to study 1, peers who were seen as popular were 1.47 times more likely to be selected than non-popular peers (see Table 3 and Fig. 4B). Similar to study 1, we found an interaction between age and friendship, however, in contrast with study 1 the odds ratio was < 1, suggesting the preference for friends may decrease with age in this sample. However, data visualisation of the predicted selection rate showed an increasing slope for friends with age, comparable with the interaction slope detected for study 1 (see Figs. 4A and 2B).

**Table 3:** Characteristics attributed to consulted peers in study 2.

	Dependent variable: consulted peer (1 = yes; 0 = no)
(intercept)	0.01, (0.01–0.01), Z = − 24.68,* p* < 0.001
Friend [= yes]	14.08, (10.71–18.50), Z = 18.96,* p* < 0.001
Most popular [= yes]	1.47, (1.11–1.95), Z = 2.65,* p* = 0.008
Trustworthy [= yes]	7.22, (5.41– 9.63), Z = 13.44,* p* < 0.001
Age (continuous)	1.51, (1.09–2.11), Z = 2.46,* p* = 0.014
Friend x Age	0.74, (0.58–0.94), Z = − 2.45,* p* = 0.014
Most popular x Age	0.92, (0.70–1.21), Z = − 0.59,* p* = 0.556
Number of participants (N classes)	278 (22)
Number of observations	7272

Linear mixed-effects models were fitted to the selected peers in the game, using ‘participant’ as random intercept. Model 1 tested whether the selected peers were friends, popular and/or trustworthy peers, and whether the selection of friends and popular peers depended on age. Odds ratios are given for the specific characteristics. The 95% confidence intervals (CI) are in parentheses, followed by* P* values. Participants who did not consult anyone are excluded from the model.

#### Adolescents also select peers perceived as likeable, admirable, cool, mean and smart

**Figure 5:**
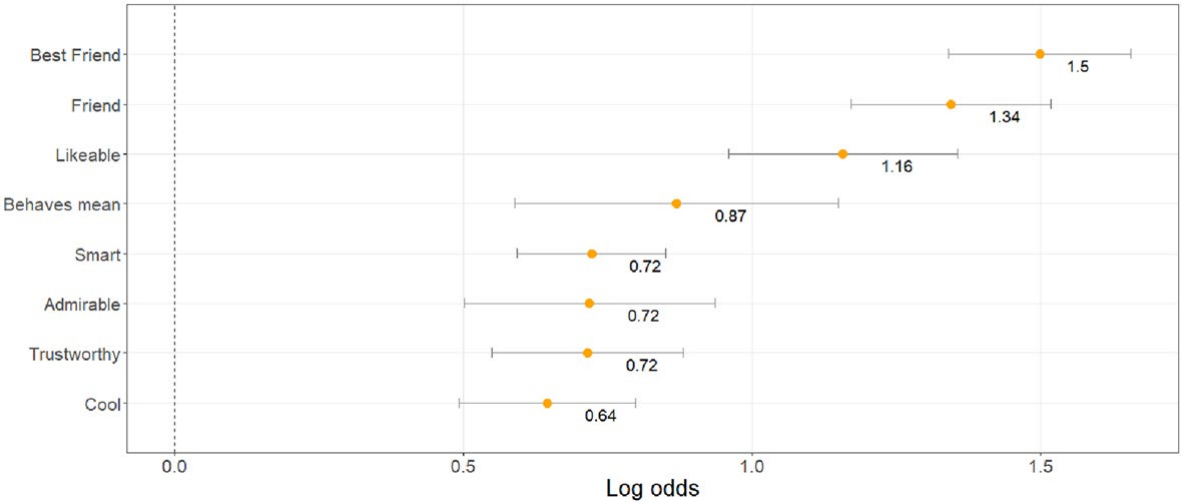
Characteristics that described selected peers in study 2 at best. Depicted are the log odds of the most important characteristics, based on the full model with a Lasso regularization for variable selection. The parameter estimates of the other characteristics were set to zero. The error bars indicate the standard error of the parameter estimates.

As in study 1, we ran a Lasso model with all peer characteristics. Variable selection, controlled by a Lasso shrinkage procedure, indicated that the best model evaluated on its AIC value included the following predictors for peer selection: seen as best friend, friend, likeable, being mean, trustworthy, smart, admirable and cool (ordered from highest to lowest effect size; see Fig. 5). See Supplemental Material Fig. S10, for the range in correlation strength between all measured peer characteristics. The characteristics ‘admirable’, ‘cool’ and ‘mean’ showed a weak correlation with being ‘most popular’. For additional details regarding the estimated coefficients of all peer characteristics, and the fit of all models across the tested shrinkage range (lambda = [0–500]), please refer to Supplemental Material Fig. S11.

## Discussion

Adolescents are often portrayed as passive receivers of social influence, where peers easily sway their decisions. To nuance this perception empirically, we show that adolescents are also motivated to gain information about the preferences of specific peers, and thus play a role in selecting the sources that may inform their decisions. In two studies we combined an experimental search paradigm with a peer nomination questionnaire, to examine what type of peer adolescents prefer to consult prior to making decisions under uncertainty. In the first study (*N* = 173, ages 11–18 y) adolescents could sequentially sample the choices from peers in their classroom social network for a gambling task. In the second study, an independent group of adolescents (*N* = 278, ages 12–17 y) was asked only once whose choices they wanted to observe from their classroom network when they would play the gambling game again. Supporting our hypothesis, we observed in both studies that friends were more often selected than other peers, and this friend bias increased with age. In contrast to our expectations, we found mixed effects for perceived popularity across our samples and analyses. While the selection of popular peers increased over age, it started out as a group of less interest to adolescents compared to non-popular peers, and finally never surpassed the importance of non-popular peers (study 1). On the other hand, in study 2 we did find a small positive effect for all ages. Furthermore, variable selection, to exploratory assess the significance of other measured peer characteristics revealed that smartness and trustworthiness were one of the most predictive characteristics for peer selection, but not popularity (study 1 & 2). Combined, these result show that while popularity has some relative importance, is it not an uniquely strong feature for predicting whom adolescents want to observe across samples.

As hypothesized, friends were consulted more often than other peers. Moreover, the Lasso Regression suggested that friendship is one of the best predictors for peer selection. These results are in line with previous studies showing a strong influence of friends on adolescent behaviour. Friends might be especially useful sources of information given their similarity in identity (also referred to as homophily^9,28^). According to social learning theory, it is rational to copy the behaviour of those that are similar in their values and goals, given that their preference-based choices likely also fit your values^9,32^. In the context of our study, it seems indeed plausible that adolescents observed mainly their friends as they felt uncertain and wanted to learn about their (risk) preference in this game prior to deciding for themselves^12,17^. When incorporating a social network measure into the analysis, we observed that selection rate decreased with network distance until distance 3: while friends (network distance = 1) were the most observed, friends of friend (distance = 2) were also more often selected than more distant peers in the network. We observed that the further away (from distance 4 onwards) the less reliable the estimates given that there are fewer observations and missing data in the friendship network might have resulted in higher distance ratings between some classmates. Alternatively, the curvilinear pattern might be explained by adolescent’s motivation to also sample choices of peers that are dissimilar and/or disliked to make sure you maintain or increase your dissimilarity with those peers. This might be especially the case for peers with no ties at all. This network distance measure allows for more precision when estimating the strength of social relationships compared to binary categorization (friends vs. non-friends), and could be useful in optimizing behavioral change interventions that target social norms set by peers^62^. Network distance has been linked to the subjective feeling of closeness between peers^30^, suggesting that transfer of information will especially happen between peers that feel closely related. Overall, the results suggest that friends and close peers are not merely influential because those are the individuals that one spends most time with and are passively observed, but rather that adolescents are motivated to find out what peers in their closest social circle would do.

An additional reason to select friends as social source is because it is likely that they can be trusted^29^. Indeed, friends, and especially best friends, were also typically seen as trustworthy. Interestingly, our Lasso Regression analysis showed that friendship and trustworthiness both explain unique variance in source selection. This suggests that other peers, who are not friends but are also considered trustworthy, are also more likely to be selected as source. This highlights the importance of epistemic trust—the need to trust the information you get to be willing to use it^63^—in adolescent selection of social sources. We also observed that friends became a stronger determinant for peer selection with age. Potentially, an increase in perceived friendship quality with age^40,64^, or the process of increased similarity due to having spent more time together might underlay this increased focus on friends with age, when selecting peers for guidance. Future studies with a broader age range (11–25 years) are needed to support whether consulting friends becomes more prominent in late adolescence, taking the length of friendship relationship into account within a longitudinal design. Moreover, along these lines, future longitudinal research should map how adolescents’ priority for obtaining popularity and maintaining good friendships changes from early to emerging adulthood(see also^65^).

Besides friendship and trustworthiness, study 2 showed that peers who were considered smart were also selected as source. The selection of smart peers is in line with the theory on social learning that suggests that following experts and successful individuals is a pervasive strategy of humans^15^, and suggests that adolescents also observe others with the desire to make a well-thought decision. In the context of our study, smart peers might be seen as those peers who would be good at figuring out whether it is worth gambling, given that smart people are generally good at performing complex cognitive operations. Indeed, calculating the odds of gaining or losing nothing involves some mathematical operations. Previous evidence for the selection of proficient models mainly stems from studies including adults and children^66–68^. Only a few studies so far suggest that adolescents take the peer’s intelligence or task-specific skills into account when choosing someone as task-partner^69^ or when choosing to adopt someone’s choice, potentially to increase one’s performance^14,30^. While perceived smartness did not survive variable selection in study 1, its limitation occurred in the final stage, and it emerged as the third strongest significant predictor in the second best model (see Table S4 and Fig. S6). Our finding on choosing smart peers as a source supports the potential adaptive nature of peer influence^11,70^, in which peers can also help adolescents make better decisions. Future research should further explore the role of ‘smartness’ as selection criterion, and should examine whether following self-selected peers improves adolescents' decision-making. This can be examined by using a task where right answers are based on the ground truth (e.g. estimation task or knowledge questions) and not on personal (risk) preferences.

Our studies provided mixed evidence for ‘perceived popularity’ as a selection criterium. First, our confirmatory models showed a small negative effect of popularity on peer selection in study 1, but a small positive effect in study 2. Additionally, in both studies, popularity was left out from the set of best predictors identified by the Lasso Regression. This indicates that perceived popularity itself is not one of the primary criteria for selecting which peer to observe. At first sight, this finding seems to contradict observational and experimental studies showing that popular peers are quite influential compared to unpopular peers^3,33,34^. However, in these experimental studies, participants had no choice than to observe these popular peers and these popular peers did not have to compete with other sources such as friends. In line with this lack of other available sources, a study found that late adolescents rather imitated an “average” peer than a peer described as being popular^69^. Taken together, this suggests that popular peers *can* be influential in some situations, but they are not always considered as useful sources to consult when competing with other available sources.

In addition, our exploratory analysis from study 2 suggests a more nuanced insight in the role of popularity. Peers who were seen as ‘cool’ and ‘admirable’ were also more likely to be selected, and this was also true for peers who were considered as acting mean (e.g., by excluding or humiliating others). These two seemingly conflicting profiles are in accordance with two distinct ways to achieve status and social influence; dominance and prestige^71^. Dominant individuals exert social influence by using intimidation and force to induce fear, whereas prestigious individuals gain influence because they are perceived as knowledgeable and successful^60,71^. Thus, our more detailed exploratory analysis suggests that specific characteristics that can be linked to these two forms of peer status (such as admirable, mean and cool) are used as selection criteria, but that perceived popularity might have had weak predictive power in both studies due to the complex multidimensional nature of this construct^57,59,60,72^.

The multidimensional conception of popular peers might also explain the inconsistent pattern that we observed for selecting popular peers across the two samples. That is, the population of study 1 was younger and in higher educational levels. There is evidence that different traits are associated with popularity across age^73,74^, gender^58,59^ and peer cultures^75^. For example, in emerging adulthood, popular peers are described as those who are central, liked and respected, whereas in adolescence, popularity is more strongly associated with ‘power’ and ‘fitting in’^73^. In addition, a recent study suggests that the behaviours that are rewarded with popularity might differ between educational tracks^76^. Thus, it might be that different types of peers were seen as popular across these samples. Future studies should follow up on specific characteristics linked to different forms of peer status, to measure to what extent (popular) peers with these characteristics impact adolescents’ attitudes and behaviours.

The design of our study allowed us to gain unique insights in the process of selecting and observing the real choices of familiar peers and allowed us to examine a broad range of peer characteristics to profile who adolescents prefer to consult. Among these strengths, there are also study constraints and related directions for future research. In our gambling task, choices relied on the adolescent risk preference (I.e., is it worth taking the risk). However, it is likely that different type of peers will be seen as valuable sources of information depending on the decision domain and the type of reward at stake. For example, popular peers might be seen as an informative source for norm following, to learn about the newest trends or which behaviours to show off to increase own status. Also, in public settings adolescents will likely attune to attend to popular peers if there is a risk of being excluded^38,77^. In contrast, friends with similar tastes will be seen as a valuable source when being uncertain about your own preferences (i.e. which social activity to engage in^32^). In addition, for matter of facts it may be wise to follow knowledgeable others, regardless of your relationship with that person or their status. Future studies should investigate multiple decision domains besides risky decision-making, such as education-related choices or consumer behaviour, to gain a better understanding of who adolescents consult in different domains. Moreover, it would also be interesting for future studies to investigate the effect of a public setting in relation to the type of peers that are followed, to gain a fuller understanding of adolescents’ motives to comply or to conform with other’s choices. It might be that peers who obtained popularity due to dominance, will be influential in public settings only, while popular peers who obtained their status due to admiration will be followed for private choices as well.

Second, our studies focused on individual peer nominations as predictors of the probability that a peer will be chosen for observation. From this perspective, we did not account for the potential interdependence of nomination data beyond their simple correlation (SI Fig. S5 and Fig. S10). In addition, in our experimental set-up participants were able to view the choices of all their classmates, regardless of their personal relationship with them. Although this provides us with important information about who adolescents prefer to observe in class, it disregards some of the structural constraints of the social network that they are in. In real life, the frequency of observing peers, and specifically the opportunity to ask for direct advice is constrained by one’s social network structure^78^ (e.g. if we are friends it is more likely that I can ask for your advice). Future studies should apply statistical models of social networks, such as Exponential Random Graphs or latent network models^79,80^. This approach can explicitly model the interdependence of nomination data and separate the processes of individual preferences and network selection effects that drive adolescents' imitation of certain peers^78,81^. Such dynamic models will also lead to a more accurate measure of social distance, as social ties in a network are not only formed by observed friendship relations but emerge from various social processes simultaneously (i.e. other matching attributes between nodes)^82^.

Finally, our studies only focused on Dutch adolescents aged 11–18 years, and their classmates as social network, who followed pre-vocational to pre-university education at high school. Thus, our results only generalize to the average demographics of our sample. Future studies could investigate potential interactions between, for example, SES, ethnicity or immigration background and peer selection. Adding these variables can help us better understand variability in peer selection within groups (e.g. peers with a similar SES are more likely to be observed for information) or between groups (e.g. comparing selection criteria for different strata of SES; cf.^83,84^). In our study the social network referred to the participants’ classmates at school only. While we are limited to providing information on who adolescents prefer to observe in class only, this focus on the class-setting alone limits the variation in access and shape of adolescents’ social network within our sample (but see^85^).

In conclusion, we provide a comprehensive view on who adolescents consult in their social networks for uncertain choices, which is an important but often overlooked aspect of peer influence. As expected, adolescents showed a bias towards observing their (best) friends, which became even more prominent with increasing age. Study 2 showed that adolescents also choose to observe smart and trustworthy peers, suggesting that adolescents use adaptive strategies when selecting information. When–admittedly speculatively—trying to extrapolate these results to an adolescent's daily life, our findings suggest that adolescents are likely to jump off a bridge if all their friends would, but that adolescents may also seek out a few smart and/or trustworthy peers that can tell them whether they would survive it. Across two studies, we find mixed results for the role of perceived popularity in peer selection. However, our data suggests at least that several characteristics that can be linked to high peer status (i.e. cool and admirable) are important for peer selection. Future studies should focus on different types of high status peers, taking these characteristics into account, and should assess whether the power of these peers is also bounded to specific social settings and age groups. Taken together, these results provide an important starting point for designing interventions that can help teens to think about their own role in selecting social sources for guidance, and challenge them to think about whether they can really learn and profit from these social sources. In this way, social influence could truly benefit adolescents’ decision-making.

### Supplementary Information

Supplementary Information.

## Data Availability

Data and code supporting the findings of this study are available from the public repository: https://osf.io/b5c9d/.
