# Supplementary material for: Identifying who adolescents prefer as source of information within their social network
Source: Sci Rep. 2023 Nov 20;13:20277. doi: 10.1038/s41598-023-46994-0 (PMC10662136; doi:10.1038/s41598-023-46994-0)
Supplement: Supplementary file 1 — Supplementary Information. [file 41598_2023_46994_MOESM1_ESM.docx]

**Supplementary Information for:**

Identifying who adolescents prefer as source of information within their social network

by

Scarlett K. Slagter, Andrea Gradassi, Anna C.K. van Duijvenvoorde, & Wouter van den Bos

Content

1. Supplementary Figures
2. Supplementary Table
3. Supplementary Results
   - - 1. **Supplementary figures**

**Fig. S1. Histogram of the number of participants for each reported age in study 1**


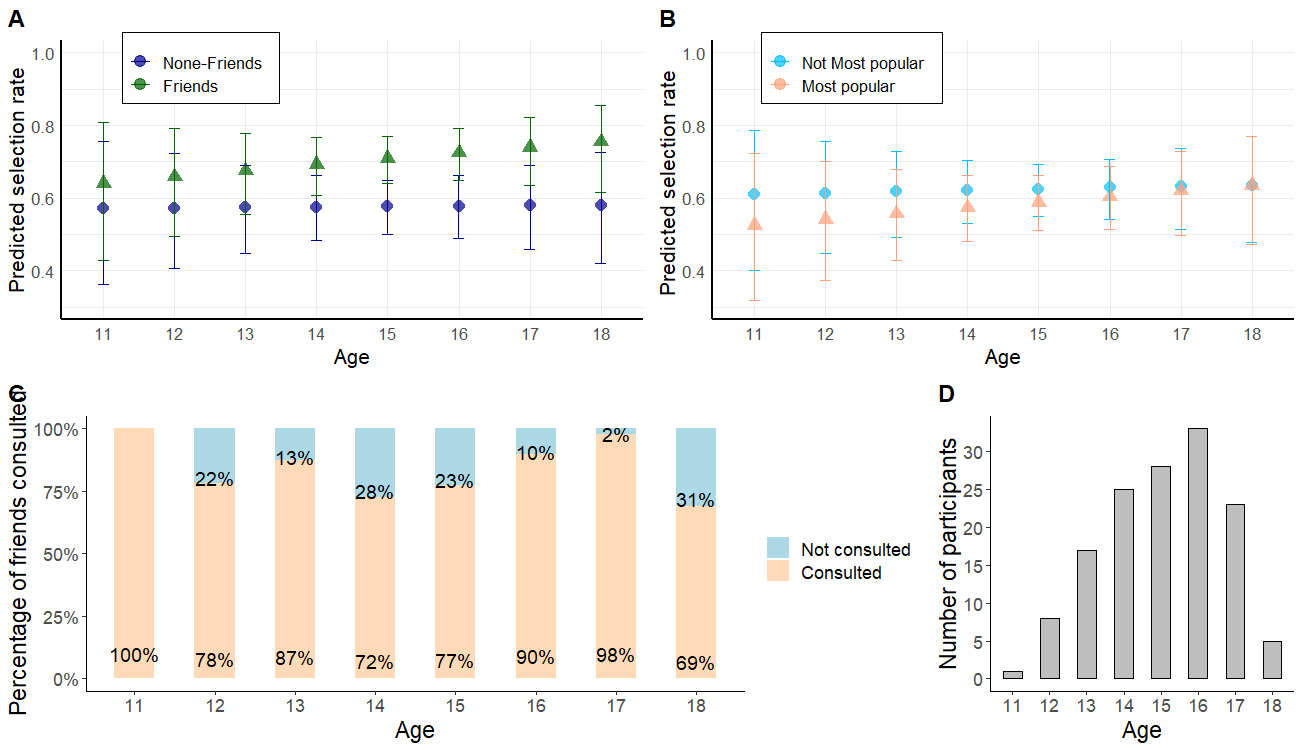


**Fig. S2. Outlook of the solo and social gambling task used in study 1**

**
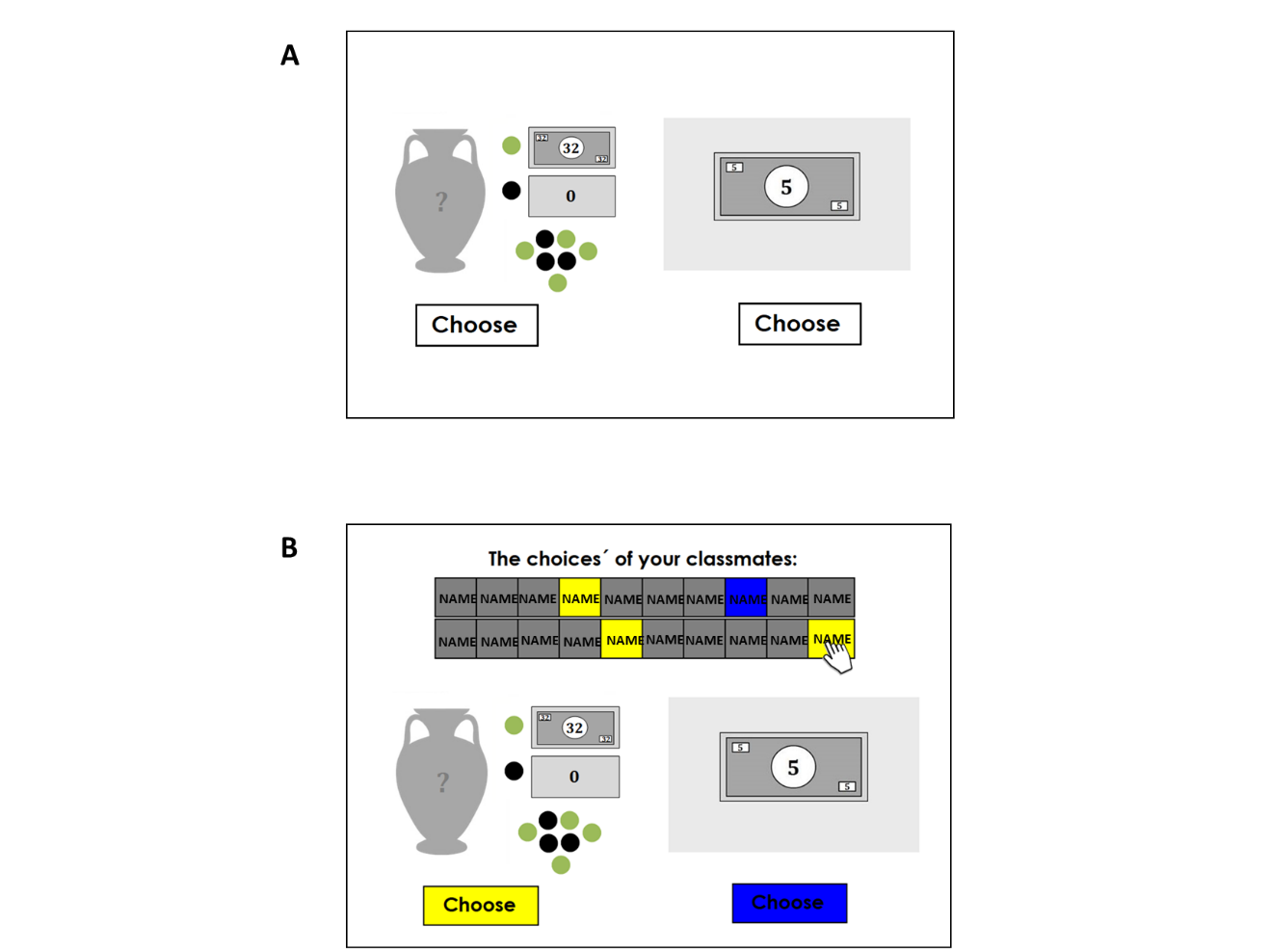
**

*Note.* (A) In the first session of study 1, participants made the gambling task on their own, without social information. Within all trials participants choose between a safe option (sure pay-off of 5 points) or a risky option (gambling vase). The risky option resulted in zero points, in case a black ball was drawn, or an amount of 8, 14, 20, 32 or 50 points (this varied per trial), when a green ball was drawn. The probability to gain when choosing the risky option was based on the distribution of green and black marbles. However, participants saw a sample of 7 marbles only to induce uncertainty about the gain probability. (B) The outlook of one trial of the solo version of the task is presented, administered to participants in a follow-up session. Here, participants could view the choices made by their classmates (obtained in session 1), for this specific trial. These choices were presented as uncovered boxes in a matrix (social board), which could be revealed by the participant by clicking on a grey box. The name of each classmate was provided on a box, to indicate the source of information.

**Fig. S3. Distribution of the search behaviour for sequential sampling of choices in study 1**


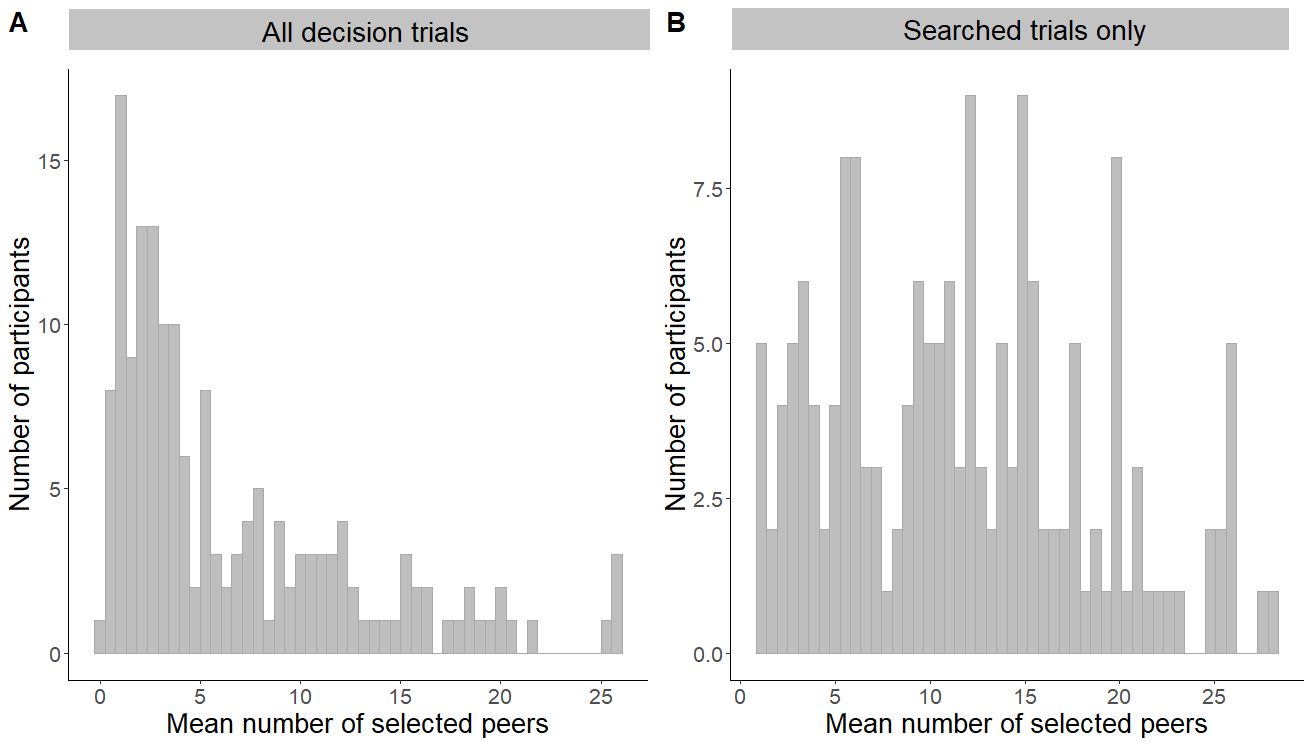

*Note.* (a) The distribution of the mean number of selected peers (i.e. sampled choices) of each participant, calculated over all 20 decision trials. (b) The distribution of the mean number of selected peers (i.e. sampled choices) of each participant, where the mean is calculated over the participant’s searched trials only. Thus, non-searched trials are excluded. (c) Displayed is the proportion of searched trials per participant, based on the total number of decision trials.

**Fig. S4. Frequency of selecting friends versus non-friends in study 2**


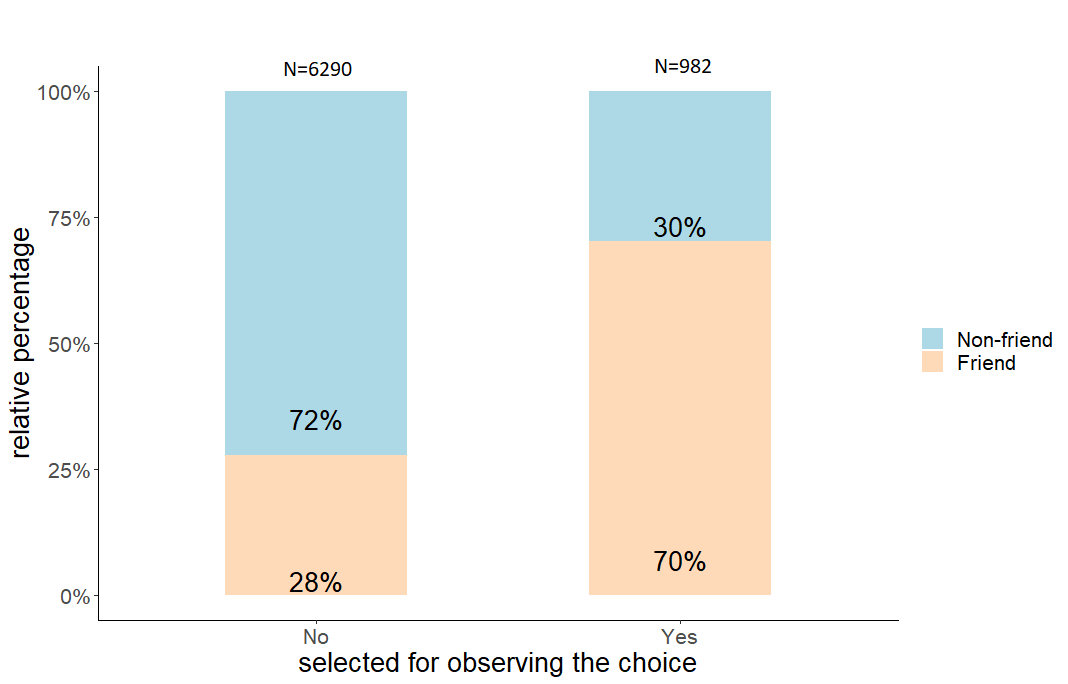


*Note.* Relative percentage of selected peers from all possible options, contrasted against whether this selected peer was nominated as a friend or not (non-friend).

**Fig S5. Strength of correlation between all peer characteristics, for which participants could nominate their classmates (study 1)**

**
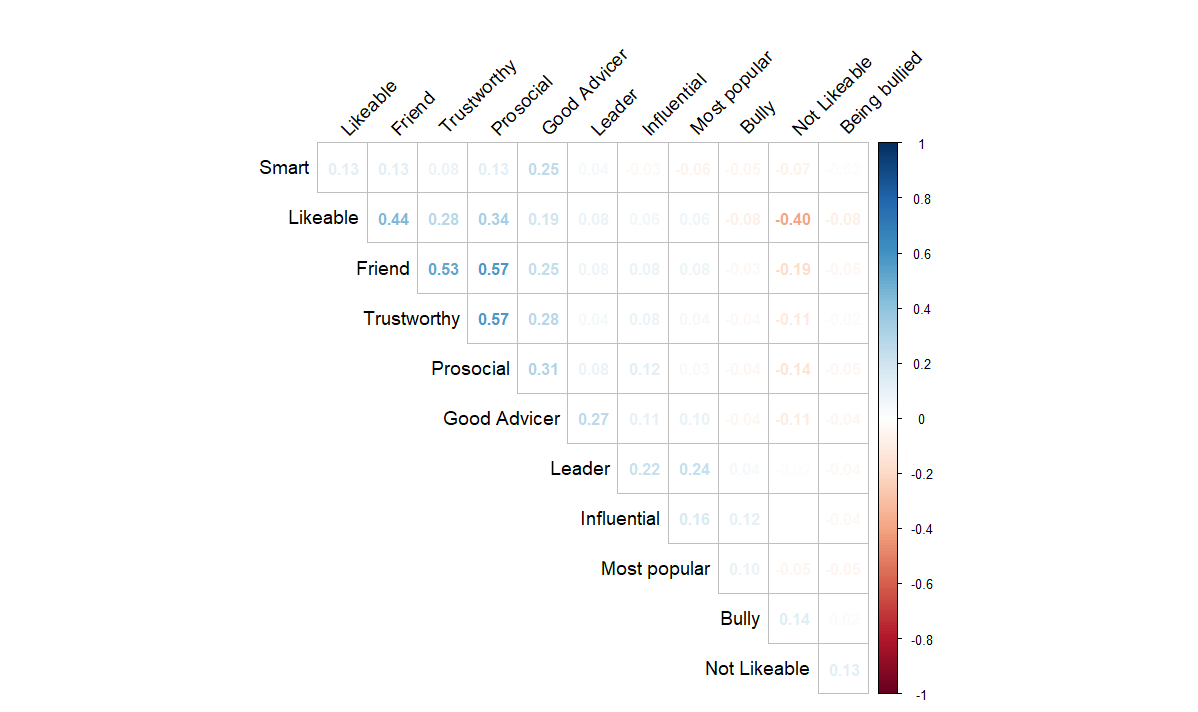
**

*Note.* Correlation matrix of all peer characteristics on which participants evaluated each classmate, by providing nominations (yes or no). The strength of the correlation is color-coded, which ranged between the -0.40 and 0.57. Only significant correlations at the significance level of 0.01 are displayed. The strongest positive relationship was between friends and pro-sociality and between prosocial and trustworthiness (both r=0.57). The strongest negative relationship was between likability and not being likable (r= -0.40).

**Fig. S6. Study 1: Overview of AIC value and estimated variable coefficients of all models across different shrinkage values**

*
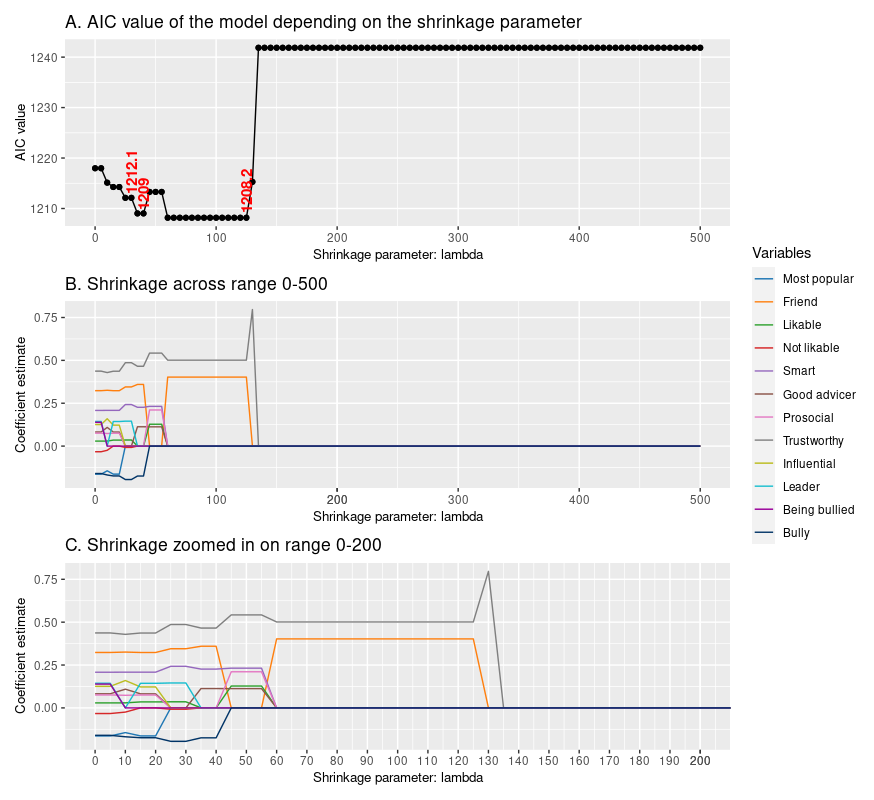
*

*Note:* The AIC values (A) and estimated coefficients of all variables (A & B) for each model are displayed as a result of the testing procedure to find the optimal shrinkage parameter (lambda) for penalization within the Lasso algorithm used for variable selection. AIC and coefficient estimates are presented for all tested lambda values, ranging from 500 to 0 in steps of 5, resulting in 100 models with different penalization factors. (A) displays the AIC values of each full model, with the top 3 lowest AIC values highlighted in red. (B) displays the shrinkage of variable coefficients (color-coded) across the applied shrinkage range of lambda=[500-0]; hence, the estimated coefficients for each tested model (n=100) can be found in Panel B. Across the tested shrinkage values, the features ‘Friend’ and ‘Trustworthiness’ are estimated as highest positive predictors. Keeping these variables resulted in the best model at lambda=[60-125], while shrinking these coefficients to zero, and thus eliminating these variables resulted in a worse model (see A). The second best performing model, at lambda=[40-35], includes smartness and good advisor as additional characteristics. The variable ‘smartness’ is estimated as third highest positive predictor in all models between the applied shrinkage range of 0-55, but eventually shrink to zero as one of the latest predictors. (C) zooms in on the lambda range 200-0 to provide a more detailed view of the changes that occurred across the tested shrinkage values for all coefficients.

**Fig. S7. Histogram of the number of participants for each reported age in study 2**

**
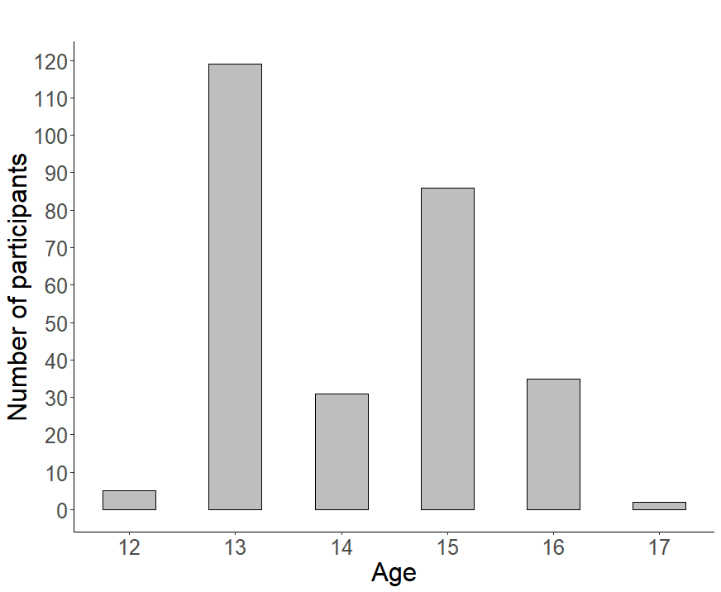
**

*Note.* Due to the COVID-19 pandemic, data-collection had to end suddenly for this study. Therefore, we were not able to recruit more participants from the age 16 and 17.

**Fig. S8. Outlook of the solo gambling task used in study 2**

**
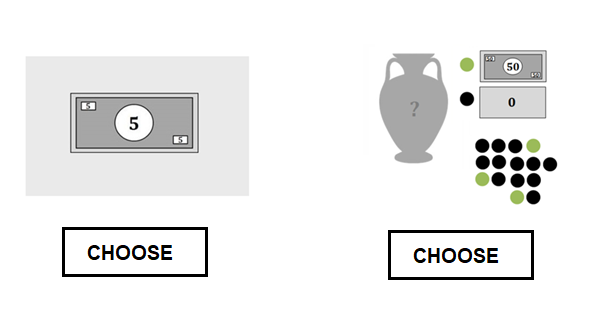
**

*Note.* An example trial of the solo version of the gambling game, used in study 2. Within each trial of the task, participants choose between a safe option (sure pay-off of 5 points) or a risky option (gambling vase). The risky option resulted in zero points, in case a black ball was drawn, or an amount of 8, 14, 20, 32 or 50 points (this varied per trial), when a green ball was drawn. The probability to gain when choosing the risky option, was based on the distribution of the green and black marbles. In this version of the game, the probability was either depicted on the vase, by showing the full distribution or by showing a sample of 3 marbles or 15 marbles from the vase. These different levels of uncertainty about the gain probability served another research goal, reported in Slagter et al. (2023).

**Fig. S9. Distribution of the search behaviour for the one-shot question of study 2**

**
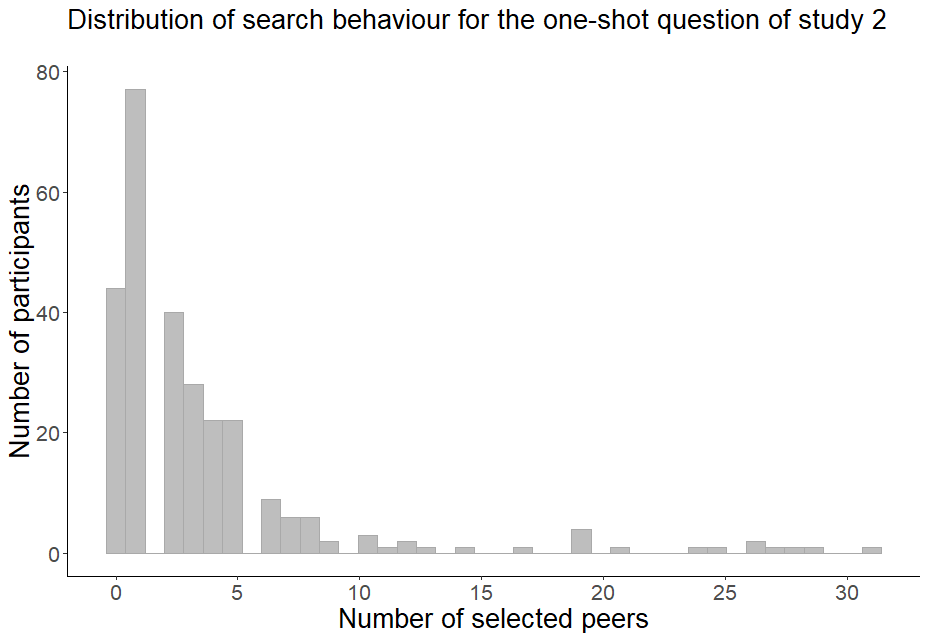
**

*Note.* The number of selected peers plotted against the frequency of participants.

**Fig. S10. Strength of correlation between all peer characteristics, for which participants could nominate their classmates (study 2)
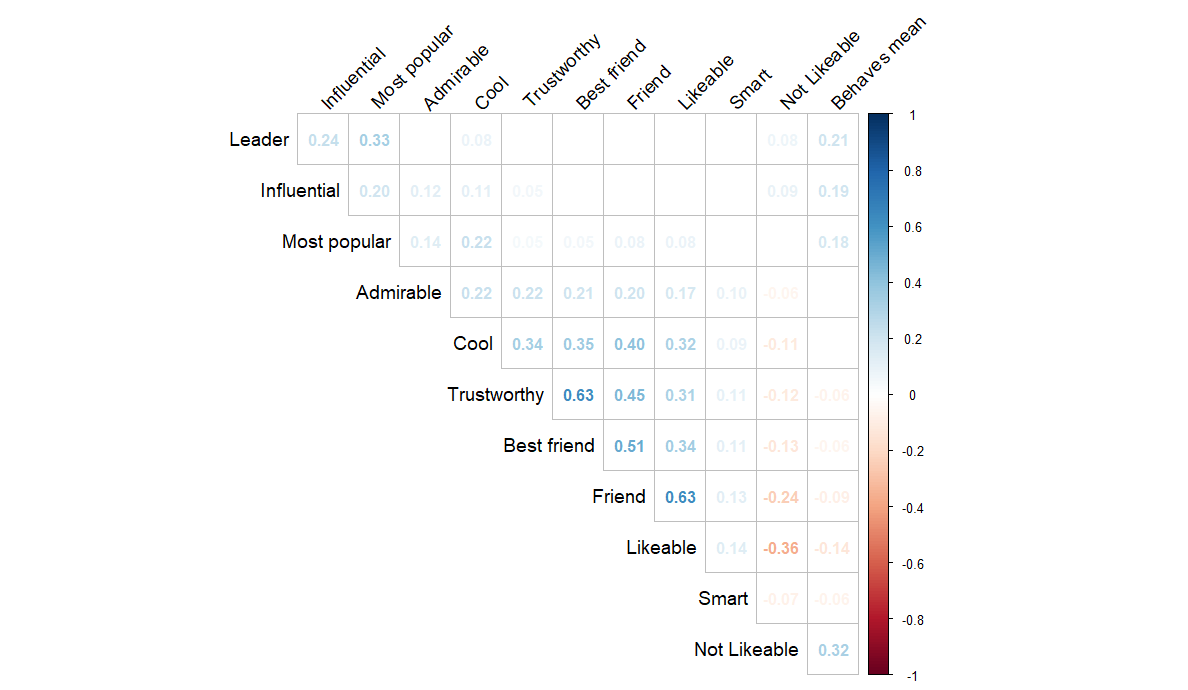
**

*Note.* Correlation matrix of all peer characteristics on which participants evaluated each classmate, by providing nominations (yes or no). The strength of the correlation is color-coded, which ranged between the -0.35 and 0.63. Only significant correlations at the significance level of 0.01 are displayed. The strongest positive relationship was between friends and likability (r(7272)= 0.63) and between best friends and trustworthiness (r(7272)= 0.63). The strongest negative relationship was between likability and not being likable. Perceived as being most popular correlated weakly with the characteristics: admirable, cool and behaving mean.

**Fig. S11. Study 2: Overview of AIC value and estimated variable coefficients of all models across different shrinkage values**


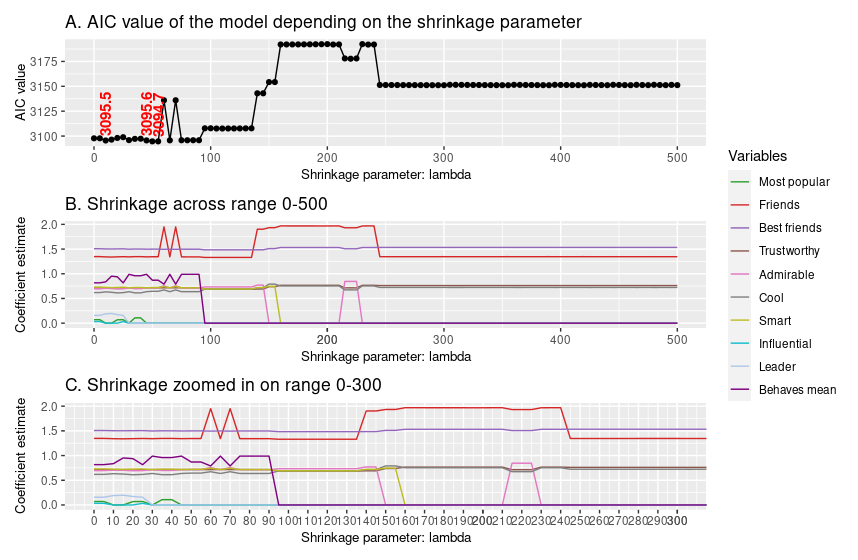


*Note:* The AIC values (A) and estimated coefficients of all variables (A & B) for each model are displayed as a result of the testing procedure to find the optimal shrinkage parameter (lambda) for penalization within the Lasso algorithm used for variable selection. AIC and coefficient estimates are presented for all tested lambda values, ranging from 500 to 0 in steps of 5, resulting in 100 models with different penalization factors. (A) displays the AIC values of each full model, with the top 3 lowest AIC values highlighted in red. (B) displays the shrinkage of variable coefficients (color-coded) across the applied shrinkage range of lambda=[500-0]; hence, the estimated coefficients for each tested model (n=100) can be found in Panel B. (C) zooms in on the lambda range 300-0 to provide a more detailed view of the changes that occurred across the tested shrinkage values for all coefficients.

- - - 1. **Supplementary Table**

**Table S1. Overview of the main model predictors tested in study 1 and 2**

| Dependent Variable:  peer selection | | | |
| --- | --- | --- | --- |
|  | Study 1: Model 1a | Study 1: Model 1b | Study 2 Model |
| Intercept | X | X |  |
| Perceived popular | X | X |  |
| Perceived as friend | X |  |  |
| Network distance |  | X |  |
| Age x popular | X | X | X |
| Age x friend | X |  | X |
| Age * Network distance |  | X |  |
| Perceived as trustworthy |  |  | X |
| Number of participants | 140 | 140 | 278 |

**Table S2. Outcome of the Network distance model of study 1**

|  | Dependent variable:  Selected peer  (Proportion of times selected in searched trials)  Model on all classes Model on subset of classes | |
| --- | --- | --- |
| Number of participants (N classes) | 140 (10) | 100 (7) |
| Number of observations | 2725 | 2126 |
| (intercept) | 1.56 (1.12 – 2.15)  Z=2.67, p=0.008 | 1.37 (0.92 – 2.05)  Z=1.55, p=0.122 |
| Network distance [=1] | 1.90 (1.74 – 2.07)  Z=14.41, p<0.001 | 1.77 (1.59 – 1.98)  Z=10.09, p<0.001 |
| Network distance [=3] | 0.83 (0.76 – 0.91)  Z=-4.03, p<0.001 | 0.85 (0.75 – 0.97)  Z=-2.37, p=0.018 |
| Network distance [=4] | 0.75 (0.67 – 0.85)  Z=-4.47, p<0.001 | 0.63 (0.52 – 0.76)  Z=-4.78, p<0.001 |
| Network distance [=5] | 0.59 (0.46 – 0.77)  Z= -3.89, p<0.001 | 0.32 (0.22 – 0.47)  Z= -5.78, p<0.001 |
| Network distance  [= No observed tie] | 1.01 (0.91 – 1.12)  Z= 0.18, p= 0.858 | 1.10 (0.96 – 1.26)  Z= 1.37, p= 0.171 |
| Network distance [=1]* Age | 1.12 (1.03 – 1.22)  Z= 2.58, p= 0.010 | 1.23 (1.08 – 1.41)  Z= 3.04, p= 0.002 |
| Network distance [=3]* Age | 1.00 (0.91 – 1.08)  Z= -0.11, p= 0.915 | 0.93 (0.79 – 1.09)  Z= -0.89, p= 0.375 |
| Network distance [=4]* Age | 1.01 (0.91 – 1.13)  Z= 0.26, p= 0.796 | 1.30 (1.00 – 1.69)  Z= -1.97, p= 0. 049 |
| Network distance [=5]* Age | 0.79 (0.59– 1.06)  Z= -1.55, p= 0.120 | 1.82 (0.91 – 3.61)  Z= -0.11, p= 0.088 |
| Network distance  [= No observed tie]* Age | 1.01 (0.91 – 1.11)  Z= 0.11, p= 0.915 | 0.83 (0.70 – 0.98)  Z= -2.19 , p= 0.029 |
| Most popular [=yes] | 0.87 (0.81 – 0.94)  Z= -3.47, p= 0.001 | 0.85 (0.78 – 0.94)  Z= -3.28, p= 0.001 |
| Most popular [=yes] *Age | 1.10 (1.03 – 1.18)  Z= 2.69, p= 0.007 | 1.14 (1.00 – 1.29)  Z= 2.01, p= 0.045 |
| Age (continuous) | 1.00 (0.73 – 1.38)  Z= -0.00, p= 0.998 | 1.16 (0.68 – 1.97)  Z= 0.54, p= 0.591 |

*Note.* In this model ‘perceived as friend’ was substituted for Network Distance, a measure related to the subjective feeling of closeness. Again, a Linear mixed-effects model was fitted to the observed proportion of times that a peer was selected in the game, using ‘participant’, nested in ‘classes’ as random intercept. The number of searched trials per participants were entered as weight in the model, giving more weight to observed selection rates of participants that searched more. The reference level was set to network distance= 2. Odds ratios are given for the specific characteristics. The 95% confidence intervals (CI) are in parentheses, followed by the Z-statistics and P values. On the left, model outcomes are reported for only the subset of data stemming from the classes that had a participant rate above 70%.

**Table S3. Overview of the participation rate per class for study 1**

| **School/ class** | **Grade** | **Level of education** | **N Participants /**  **class size** | **% participation** |
| --- | --- | --- | --- | --- |
| 1/ 1 | 3 | VMBO | 19/20 | 95 |
| 1/ 2 | 5 | VWO | 29/31 | 94 |
| 2/1 | 1 | VWO | 13/17 | 76 |
| 2/2 | 1 | VWO | 20/28 | 71 |
| 2/3 | 3 | HAVO | 13/25 | 52 |
| 2/4 | 3 | HAVO | 12/19 | 63 |
| 2/5 | 3 | VWO | 16/29 | 55 |
| 2/6 | 3 | VWO | 21/28 | 75 |
| 2/7 | 5 | VWO | 27/29 | 93 |
| 2/8 | 5 | VWO | 21/23 | 91 |

**Table S4. Top 3 best models based on AIC criteria when finding the most optimal shrinkage parameter for study 1**

|  | **AIC value** | **Value of lambda** | **Subset of variables** |
| --- | --- | --- | --- |
| **Winning model** | 1208.2 | [60-125] | Friendship (0.40**)  Trustworthiness (0.50**) |
| **Second best model** | 1209.0 | [40-35] | Friend (0.36**) Trustworthy (0.46**)  Smartness (0.23*)  Good advisor (0.11)  Bully (-0.17) |
| **Third best model** | 1212.1 | [30-25] | Friend (0.35**) Trustworthy (0.49**)  Smartness (0.24*)  Leader (0.15)  Bully (-0.20)  Likeable (0.04)  Not likeable (-0.01) |

*Note.* Overview of the top 3 best fitting models that resulted from the variable selection procedure, for which 100 models with a different shrinkage parameter (lambda ranged between 0-500 in steps of 5) were tested. Only non-zero variables, that survived shrinkage, are presented in the table. The coefficient estimate (beta) are in parenthesis, with the significant predictors marked by: ** = p<0.005, * = p<0.05.

- - - 1. **Supplementary Results**

**Section 1: Use of revealed information in Study 1**
Our main results indicated that adolescents search for social information from specific individuals. As a follow-up, we explored whether participants used their sampled information from others in subsequent decision making. However, note that the study was not designed for measuring the impact of the revealed information, as we did not control for the type and amount of information participants revealed. A logistic mixed-effects model was used to predict participant’s final decision ( risk or safe) by means of the sampled social information (i.e. classmates’ choices). The revealed social information was entered as the proportion of opened boxes in favour of risk (i.e. proportion of risky choices). We accounted for the participant’s preference to choose risk, by adding the initial solo choices of the participant, that had been made on this same set of trials during the solo session (session 1). Only trials in which participants revealed choices were included, and one participant was excluded as outlier based on its risky choice proportion of 1. This resulted in 1758 observations to be modelled stemming from 163 participants (9 participants did not search for the choices of others during the game).

Indeed, an increase in the proportion boxes in favor of risk, increases the odds of choosing risk ( OR=4.13, 95% (3.41 – 5.02), p<0.001) as final choice in the social version. This is beyond the prediction based on the participant’s initial solo choice on that specific trial, where choosing risk showed to be 7.52 times more likely when participant’s previously choose risk as well (OR =8.53, 95% (6.34 – 11.47), p<0.001).

**Section 2**
**Additional information on the decrease in selection rate only apparent until distance 3.**

We observed that the gradual decrease in selection rate was apparent until distance 3 (peers related to the friends of their friends), but stagnated at distance 4. This observation might occurred as network distances above 3 might reflect the occurrence of missing data rather than real social distance. In addition, the none-category, consisting of unconnected peer dyads is challenging to interpret, as the social distance between these peers could not be established due several reasons (e.g. missing data, no reciprocal nominations).

The none category indicates participants that were not connected through a chain of friends, and thus suggest to be really distant. However, given that in each classroom there were some students that did not participate this resulted in some missing data (See Table S1), with three classes below the recommend participation rate to construct a friendship network (Neal, 2020). As a result, this could have led to missing bridge ties between different clusters of the network, which connects peers of different friend groups, and thus in reality these friend groups are less socially distant. Moreover, friendship ties were only created when friendship nominations were reciprocal. However, not reciprocal relationships were observed in our data as well. In those cases, one peer felt more closely connected to the other peer in the dyad than vice versa.
